# Benzene exposure is associated with cardiovascular disease risk

**DOI:** 10.1371/journal.pone.0183602

**Published:** 2017-09-08

**Authors:** Wesley Abplanalp, Natasha DeJarnett, Daniel W. Riggs, Daniel J. Conklin, James P. McCracken, Sanjay Srivastava, Zhengzhi Xie, Shesh Rai, Aruni Bhatnagar, Timothy E. O’Toole

**Affiliations:** 1 Diabetes and Obesity Center, University of Louisville, Louisville, Kentucky, United States of America; 2 Institute of Molecular Cardiology, University of Louisville, Louisville, Kentucky, United States of America; 3 Department of Environmental and Occupational Health Sciences, University of Louisville, Louisville, Kentucky, United States of America; 4 Department of Bioinformatics and Biostatics, University of Louisville, Louisville, Kentucky, United States of America; 5 Biostatistics Shared Facility, JG Brown Cancer Center, University of Louisville, Louisville, Kentucky, United States of America; Hokkaido Daigaku, JAPAN

## Abstract

Benzene is a ubiquitous, volatile pollutant present at high concentrations in toxins (e.g. tobacco smoke) known to increase cardiovascular disease (CVD) risk. Despite its prevalence, the cardiovascular effects of benzene have rarely been studied. Hence, we examined whether exposure to benzene is associated with increased CVD risk. The effects of benzene exposure in mice were assessed by direct inhalation, while the effects of benzene exposure in humans was assessed in 210 individuals with mild to high CVD risk by measuring urinary levels of the benzene metabolite *trans*,*trans*-muconic acid (*t*,*t*-MA). Generalized linear models were used to assess the association between benzene exposure and CVD risk. Mice inhaling volatile benzene had significantly reduced levels of circulating angiogenic cells (Flk-1^+^/Sca-1^+^) as well as an increased levels of plasma low-density lipoprotein (LDL) compared with control mice breathing filtered air. In the human cohort, urinary levels of *t*,*t*-MA were inversely associated several populations of circulating angiogenic cells (CD31^+^/34^+^/45^+^, CD31^+^/34^+^/45^+^/AC133^–^, CD34^+^/45^+^/AC133^+^). Although *t*,*t*-MA was not associated with plasma markers of inflammation or thrombosis, *t*,*t*-MA levels were higher in smokers and in individuals with dyslipidemia. In smokers, *t*,*t*-MA levels were positively associated with urinary metabolites of nicotine (cotinine) and acrolein (3-hydroxymercapturic acid). Levels of *t*,*t*-MA were also associated with CVD risk as assessed using the Framingham Risk Score and this association was independent of smoking. Thus, benzene exposure is associated with increased CVD risk and deficits in circulating angiogenic cells in both smokers and non-smokers.

## Introduction

Benzene is a ubiquitous environmental pollutant. In the United States, it is one of the top 20 chemicals produced by industrial sources, which yearly release over 6.7 million pounds of benzene into the air [1,2]. Substantial amounts of benzene are also generated by mobile sources, and together, these emissions deliver high levels of atmospheric benzene, especially near point sources [3–5]. Humans are also exposed to benzene generated by the combustion of organic material, as found in mainstream or secondhand cigarette smoke. Mainstream cigarette smoke contains 35–70 ppm benzene, and even higher concentrations of benzene are generated from other tobacco products such as water pipes, cigars and pipe tobacco [6,7]. Therefore, humans are frequently exposed to high levels of benzene generated from indoor and outdoor sources [8].

Though benzene is present at high concentrations in air pollutants known to increase CVD risk, little is known about the cardiovascular effect of benzene per se. Cardiovascular tissues are highly sensitive to inhaled pollutants and several studies have shown that inhalation of toxic substances such as cigarette smoke and particulate matter (PM_2.5_) cause significant cardiovascular injury [9–13]. Indeed, myocardial infarction and stroke are the leading causes of death in smokers, and more than 70% of excessive premature mortality associated with ambient particulate air pollution is due to cardiovascular causes [9]. Hence, we measured urinary levels of the benzene metabolite *trans*, *trans*-muconic acid (*t*,*t*-MA) in individuals with mild to high CVD risk and examined associations with traditional CVD risk factors and circulating angiogenic cell populations, which are reported to maintain vascular integrity and predict CV events and mortality [14–16]. Our results show that benzene exposure is associated with a suppression of circulating angiogenic cells (CACs), cells that have been found to be sensitive to inhaled pollutants and are predictive of cardiovascular events and increased CVD risk in humans. A suppression of CAC levels was also observed in mice exposed directly to benzene, supporting the biological plausibility of a direct effect of benzene on circulating CAC levels. Taken together, these observations support that notion that exposure to benzene could result in significant cardiovascular injury and increase the risk of developing cardiovascular disease.

## Materials and methods

### Murine inhalation exposure

Male C57BL/6 mice (10 weeks old, n = 10/treatment) were purchased from the Jackson Laboratory and housed in AALAC- and USDA-accredited facilities at UofL. The animals had access to food and water *ab libitum*, except during exposure periods. Benzene atmospheres were generated from a certified permeation tube system (Kin-Tek, LaMarque, TX) into a custom exposure chamber (Teague Enterprises, Inc.) as previously described for acrolein [17]. Exposures to benzene (50 ppm) or HEPA-filtered air were for 6 hours/day x 5 days/week x 6 weeks. Mice were euthanized by *i*.*p*. injection with pentobarbital at a dose of 150mg/kg. After injection, the animals were placed in a clean cage until they lacked response to physical stimuli, which was approximately 8 minutes post-injection. All procedures were approved the University of Louisville IACUC 16688.

### Human study population

Individuals (>18 years of age) with mild to high CVD risk were recruited from the University of Louisville Hospital and affiliated clinic system between October 2009 and March 2011. All accessible patients (nearly 900 individuals) visiting the clinics during this time period were pre-screened through medical records review prior to recruitment to exclude individuals that did not meet the enrollment criteria. In addition, persons unwilling or unable to provide informed consent or with significant and/or severe comorbidities were excluded. Exclusion criteria included: significant chronic lung, liver, kidney, hematological, or neoplastic disease, chronic neurological or psychiatric illness, chronic infectious disease such as HIV or hepatitis, severe coagulopathies, drug/substance abuse, and chronic cachexia. Pregnant women, prisoners, and other vulnerable populations were also excluded from the study. Patients who met the enrollment criteria (n = 210) and gave written consent were consented and administered a questionnaire (S1 File), which included demographic information, residential address, smoking status and history, secondhand smoke exposure, alcohol consumption, physical activity status, medication usage, and CVD history including incidence of heart attack, heart failure, angina, hypertension, hypercholesterolemia, diabetes, stroke, revascularization, arrhythmia, peripheral artery disease, aortic aneurism, and bleeding disorders. Medical records were reviewed to verify data obtained from subject interview. Median household income was designated at the U.S. Census Bureau block group geographic level. The study was approved by the University of Louisville Institutional Review Board (IRB 09.0174).

### Sample collection and analysis

Murine blood was collected by cardiac puncture after euthanasia. In mice, circulating levels of Flk-1^+^/Sca-1^+^ cells were measured by flow cytometry [15]. Fasting plasma levels of low-density lipoprotein (LDL) and high-density lipoprotein (HDL) were measured on the Cobas Mira 5600 Autoanalyzer using commercially available kits (Sekisui Diagnostics). Human blood was obtained from participants at the time of visit and 15 phenotypically distinct populations were quantified by flow cytometry [14]. Platelet-leukocyte aggregates were identified by flow cytometry and quantified as a percentage of total events double positive for CD41^+^ and CD45^+^ [14, 15]. Fibrinogen (STA Fibrinogen Kit), and C-reactive protein (VITROS kit) were measured on the Cobas Mira.

### Measurement of urinary metabolites

The concentration of *t*,*t-*MA in the urine was measured by GC/MS using modifications of a previously described method [18]. Briefly, 1.0 nanomole of [D4] muconic acid (CDN isotopes) was added to 0.5mL urine in an 8mL glass vial. After addition of 50μL of HCl, the mixture was extracted with 1mL ethyl acetate. Sodium sulfate was added to the ethyl acetate layer to remove excess water. The ethyl acetate was then transferred to a 2mL vial and dried under N_2_. The *t*,*t*-MA was derivatized by the addition of 100μL of acetonitrile and 50μL of BSTFA, and the sample was heated at 60^o^ C for 30 min. The derivatized sample was injected directly in the GC (Agilent Technologies, 6890 N), equipped with a mass detector (Agilent Technologies 5973). The concentration of *t*,*t*-MA in the sample was calculated from the ratio of TMS-muconic acid (m/z 271) and [D4]TMS-muconic acid (m/z 275). Urinary levels of *t*,*t*-MA were normalized to creatinine, which was measured using a commercial kit (Thermo Fisher, Infinity Creatinine Kit) with a Cobas Mira 5600 Autoanalyzer.

Cotinine was measured using a GC/MS (Agilent Technologies) method [19]. One mL of urine from self-reported current non-smokers, or 0.25 mL of urine from self-reported current smokers (diluted with 0.75 mL of deionized water) was pipetted into Teflon vials. Either 0.2 nmoles (non-smokers) or 0.5 nmoles (smokers) of the internal standard of D-3 cotinine in 0.175 mL of methanol was added followed by the addition of 0.05 mL of 0.1M NaOH and 0.325 mL of chloroform. This mixture was then centrifuged at 13000 rpm for 4 min. After discarding the aqueous layer, 100 mg of sodium sulfate was added to remove excess water, briefly mixed, and the solution was allowed to sit at room temperature for 1 min. The clear organic extract was transferred to a gas chromatography vial and 0.001 mL was injected to the GC. The *m/z* values of ions used to monitor cotinine were: 176 (cotinine) and 179 (D-3 cotinine).

Urinary levels of 3-HPMA were quantified using GC/MS as previously described [14]. For each assay, 1 mL urine was combined with 2.5 nmol of ^13^C HPMA (internal standard) and added to an Oasis Max Solid Phase Extraction column for purification. The column protocol included sequential application of: 6 mL MeOH, 6 mL 2% NH_4_OH, urine, 6 mL 2% NH_4_OH, and 6 mL MeOH. The column was then dried with N_2_, and washed with 6 mL 2% formic acid and eluted with 30% MeOH in 2% formic acid. The 3-HPMA fraction was lyophilized and reconstituted in 1 mL water. The solution was then syringe-filtered and purified using HPLC. The 3-HPMA fraction was lyophilized and subsequently derivitized with 40 μL acetonitrile and 40 μL N,O-Bis(trimethylsilyl)trifluoroacetamide (BSTFA) for 1 h at 60^°^C. One μL of the sample was applied to GC/MS (Agilent 6890N) for quantification. The ion fragments 366 (3-HPMA) and 369 (C^13^ HPMA) were compared for quantification. The values of 3-HPMA were normalized to creatinine.

### Statistical analyses

In the human study, the primary exposure variable, urinary *t*,*t*-MA, was examined for association with the primary outcome variable (CAC counts) and secondary outcome variables (e.g. CVD risk factors, fibrinogen). The levels of *t*,*t*-MA in the urine were stratified into tertiles of high, medium, and low values based on the distribution of the *t*,*t*-MA values for the entire cohort. For discreet categorical predictor variables (i.e. gender, medication history) frequencies and percentages were computed and compared among the primary exposure variable tertiles using the Chi Square test. For the continuous predictor variables, mean and standard deviation values were compared amongst the *t*,*t*-MA tertiles using ANOVA. Due to higher variability in continuous predictor variables (i.e. hsCRP, fibrinogen), p-values were calculated from log transformed data. Predictors associated at p<0.05 level with the exposure variable were considered potential confounders and corrected for in the final models.

Generalized linear models were used to examine whether the levels of CACs were associated with urinary *t*,*t-*MA levels, after adjusting for specific variable (ethnicity, HLD and cotinine). CAC levels appeared to follow the gamma distribution; therefore, generalized linear models that assessed circulating angiogenic cells as the dependent variable utilized the gamma probability distribution and the log link function. In preliminary analysis, we examined the association of 15 different populations of CACs with *t*,*t*-MA values. Four of these populations were highly correlated with urinary *t*,*t*-MA levels, and were therefore selected for further analysis.

**Fig 1:**
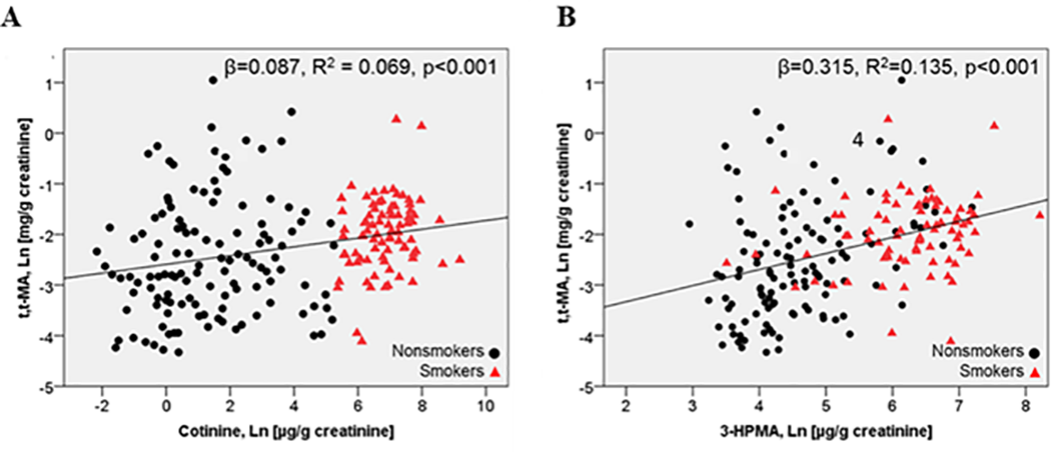
Association between cigarette smoke exposure and *t*,*t-*MA levels. Associations of *t*,*t*-MA with cotinine (A) and 3-HPMA (B) were established by regression analysis. Cigarette smoke is the major source of benzene exposure in the smoking sub-group.

Subset analyses (e.g. non-smokers, ethnicity) determining CAC and *t*,*t*-MA associations were also performed. Significant other predictors at α = 0.05 were included in the preliminary model. However, the final model contains only predictors that remained significant at five percent. These demographic groups displayed associations with the primary exposure variable, and therefore needed further analysis to determine unique relationships to the primary outcome variable (CACs). The non-smoker population was adjusted for ethnicity, hyperlipidemia and cotinine while ethnic groups (African Americans and Whites) were adjusted for hyperlipidemia and cotinine based on results from Table 1. Given that the non-smokers and different ethnic groups had different demographic associations with *t*,*t*-MA for each CAC population, a subset analysis was performed to interrogate the relationship between CACs and *t*,*t*-MA in the context of race in Table 2. To assess the contribution of benzene exposure from smoking we regressed *t*,*t-*MA against common metabolites of cigarette smoke exposure (cotinine and 3-HPMA) in Fig 1. To further graphically display the difference between smokers and non-smokers in Fig 1, we utilized the commonly used cut-point of 200 μg cotinine/g creatinine–where individuals with cotinine levels below 200 μg cotinine/g creatinine were considered non-smokers. These cut-points generated from the cotinine data were used in Fig 1B when displaying the relationship between *t*,*t*-MA levels and 3-HPMA.

**Table 1:** Demographics and CVD risk history stratified by *t*,*t*-MA.

**Categorical Variable–n (%)**	**Total** **n = 210**	**Low** ***t*,*t*-MA** **n = 70**	**Medium** ***t*,*t-*MA** **n = 70**	**High** ***t*,*t-*MA** **n = 70**	***P* value**	
**Gender**					0.440	
Female	101 (48)	32 (46)	38 (54)	31 (44)		
Male	109 (52)	38 (54)	32 (46)	39 (56)		
**Ethnicity**					0.001	*
White	118 (56)	26 (37)	43 (61)	49 (70)		
African American	88 (42)	42 (60)	27 (39)	19 (27)		
Hispanic/Latino	4 (2)	2 (3)	0 (0)	2 (3)		
**CVD Risk Factors**						
Hypertension	168 (81)	58 (84)	57 (83)	54 (78)	0.677	
Hyperlipidemia	128 (63)	39 (58)	37 (54)	52 (75)	0.021	*
Diabetes	54 (26)	18 (26)	19 (28)	17 (25)	0.928	
Obese	118 (58)	38 (57)	40 (59)	40 (58)	0.969	
Current smoker (self-report)	82 (39)	16 (23)	33 (48)	33 (47)	0.003	*
Never smoked (self-report)	57 (27)	25 (36)	16 (23)	16 (23)	0.132	
Former smoker (self-report)	69 (33)	28 (40)	20 (29)	21 (30)	0.311	
Environmental smoke (self-report)	38 (30)	10 (19)	13 (37)	15 (41)	0.053	
High CVD risk category	166 (79)	48 (69)	59 (84)	59 (84)	0.031	*
**Medical History**						
Myocardial infarction	70 (34)	21 (30)	24 (35)	25 (36)	0.782	
Stroke	20 (10)	7 (10)	3 (4)	10 (14)	0.137	
CABG/ PCI/ Stents	56 (27)	14 (20)	17 (25)	25 (36)	0.107	
Heart failure	37 (18)	12 (18)	12 (17)	13 (19)	0.972	
**Medication**						
ACE inhibitor	112 (55)	35 (51)	40 (59)	37 (54)	0.635	
Angiotensin-receptor blockers	12 (6)	5 (7)	4 (6)	3 (4)	0.779	
Aspirin	116 (57)	35 (51)	40 (59)	41 (60)	0.476	
Beta-blocker	129 (63)	41 (59)	44 (65)	44 (65)	0.760	
Calcium-channel blockers	46 (22)	14 (20)	17 (25)	15 (22)	0.801	
Diuretics	79 (39)	25 (36)	31 (46)	23 (34)	0.330	
Statins	108 (53)	31 (45)	34 (50)	43 (63)	0.086	
Vasodilator	47 (23)	16 (23)	20 (29)	11 (16)	0.185	
**Continuous Variable–mean (SD)**	**Total**	**Low** ***t*,*t*-MA**	**Medium *t*,*t*-MA**	**High** ***t*,*t*-MA**	***P* value**	
**Age** (years)	51 (10)	52 (11)	49 (10)	52 (10)	0.247	
**Cotinine** (μg/g creatinine)	521 (1050)	146 (373)	686 (1431)	725 (963)	<0.001	*
**Framingham Risk Score (FRS)**	8 (8)	7 (7)	7 (7)	12 (9)	0.259	
**Lymphocyte count X 10**^**4**^	13 (9)	13 (9)	13 (9)	14 (10)	0.724	
**Thrombosis**						
Fibrinogen (mg/dL)	346 (109)	355 (128)	345 (111)	339 (82)	0.778	
Platelet-leukocyte aggregates	11 (6)	11 (5)	10 (5)	11 (7)	0.718	
**Inflammation**						
hsCRP (mg/L)	5 (5)	5 (5)	4 (4)	5 (5)	0.590	
**Median household income** (X $1000 USD)	31 (18)	29 (18)	35 (20)	30 (16)	0.066	

Tertiles are based on log transformed *t*,*t*-MA values. Ranges for the tertiles for low, medium and high *t*,*t*-MA levels are 0.0132–0.0615mg/g creatinine, 0.0625–0.155mg/g creatinine and 0.159–2.85mg/g creatinine, respectively. Individuals with a body mass index ≥ 30 were considered obese. Current, never, and former smokers were identified on the basis of self-reported smoking status. Individuals with high FRS category had a FRS ≥ 20 or had previously experienced a cardiovascular event. Vasodilators include nitrates and hydralazine. Platelet-leukocyte aggregates are defined as the percent of CD41^+^/CD45^+^ events. The sum of CVD risk factors includes the following: Framingham risk factors: age ≥ 40 years, male gender, current smoker, hypertension, hyperlipidemia, and diabetes. Median household income is in United States dollars (USD), at the US Census block group level. CABG, coronary artery bypass graft; PCI, percutaneous coronary intervention; ACE, angiotensin-converting-enzyme; hsCRP, high sensitivity C-reactive protein

* p<0.05.

**Table 2:** Association between *t*,*t*-MA and CACs.

*t*,*t*-MA Regression	Cell type-2 (CD31^+^/34^+^/45^+^)	Cell type-8 (CD31^+^/34^+^/45^+^/AC133^–^)	Cell type-11 (AC133^+^)	Cell type-14 (CD34^+^/45^+^/AC133^+^)
**Total Population**, *adjusted for ethnicity*, *hyperlipidemia*, *and cotinine* n = 210				
Change (%)	-8.739	-8.923	-0.4687	-8.936
*P* value	< 0.001*	<0.001*	0.870	<0.001*
**Non-smokers**, *adjusted for ethnicity*, *hyperlipidemia*, *and cotinine* n = 128				
Change (%)	-9.359	-9.365	-2.621	-8.955
*P* value	<0.001*	<0.001*	0.364	<0.001*
**White**, *adjusted for hyperlipidemia*, *and cotinine* n = 118				
Change (%)	-8.883	-8.848	-3.363	-8.405
*P* value	0.002*	0.002*	0.068	0.016*
**African American**, *adjusted for hyperlipidemia and cotinine* n = 88				
Change (%)	-6.770	-7.239	5.753	-9.973
*P* value	0.253	0.212	0.008*	<0.001*

Change represents percent change per 0.1mg *t*,*t-*MA/g creatinine.

*: p<0.05

To adequately describe the phenotype of the population in relation to CVD, the population was dichotomized into low and high risk strata. An FRS below 20 was considered low risk. Individuals with FRS above 20 or those receiving secondary preventive care were assigned to the high risk category. Differences in mean *t*,*t*-MA values among the risk strata were compared using a two-sample *t*-test analysis. To evaluate the association between cigarette-independent benzene exposure and CVD risk, only the non-smokers in the high risk category were examined. For data management and statistical analyses, the IBM SPSS Statistics version 21.0 for Windows was used. For our murine exposure data analysis, the levels of Flk-1^+^/Sca-1^+^ cells (CACs), LDL and HDL mice were compared with the HEPA-filtered air control mice using a two sample t-test analysis.

## Results

### Cardiovascular effects of benzene inhalation in mice

**Fig 2:**
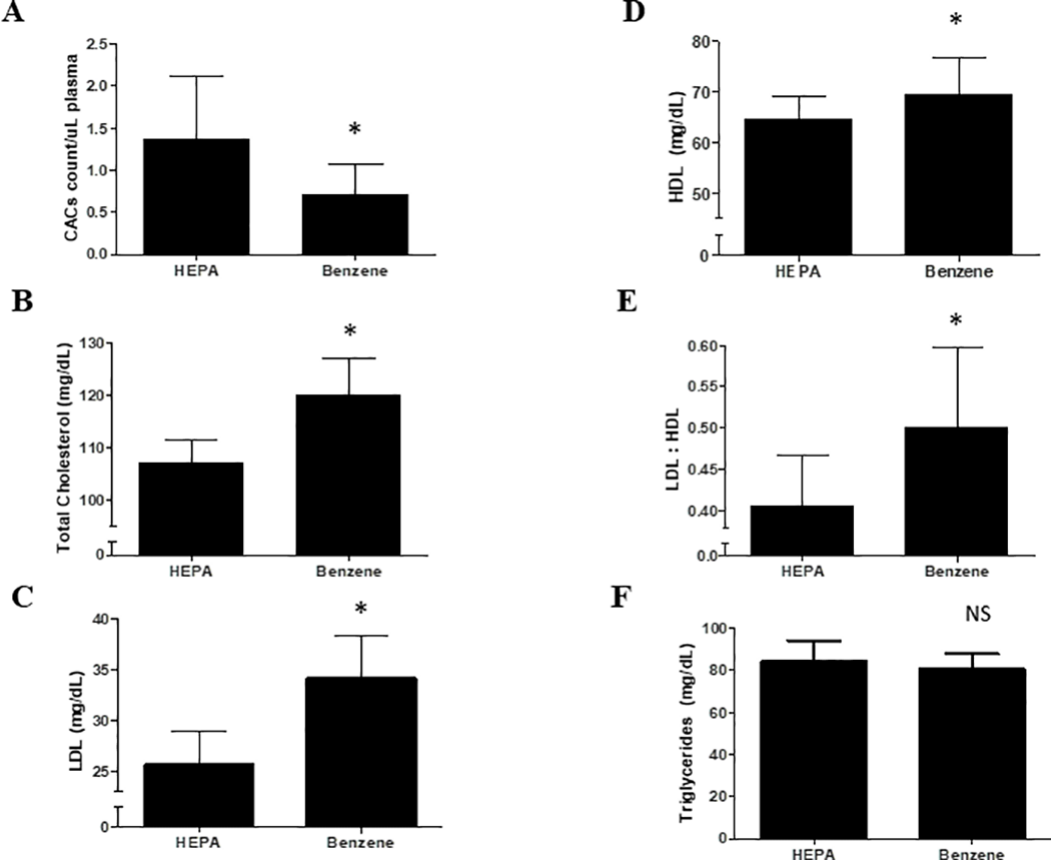
Volatile benzene exposure decreases murine CACs and increases lipoprotein levels. (A) Flk-1^+^/Sca-1^+^ cells were measured in C57Bl/6 mice after 6 weeks of exposure to volatile benzene (50ppm, 6h/d) and filtered air. The benzene exposed animals showed an approximate 48% decrease in these cells after six weeks of benzene exposure. Levels of total plasma cholesterol (B), LDL (C), HDL (D), triglycerides (F) were also quantified in the exposed mice. An LDL:HDL ratio was also calculated (E). All plasma variables except triglycerides were elevated in benzene-exposed mice compared with the filtered air exposed controls. n = 22-39/treatment group; *: p<0.05.

While the carcinogenic [20, 21] and hemato-toxic [22–24] effects of benzene are known, it is not clear if exposure to this aromatic also contributes to cardiovascular disease. To begin to examine this, we first assessed the level of circulating angiogenic cells (Flk^+^/Sca^+^) in mice inhaling volatile benzene. Levels of Flk^+^/Sca^+^ cells serve as a suitable indicator of vascular health as pauperization of analogous cells in humans is associated with deficits in vascular repair and is predictive of cardiovascular event and mortality. We found that, in mice exposed to benzene, Flk-1^+^/Sca-1^+^ cells were significantly suppressed (p = 0.002). In comparison with mice inhaling filtered air, those mice exposed to benzene had, on average, 3.4% lower levels of Flk-1^+^/Sca-1^+^ cells per 1.0 mg *t*,*t*-MA/g creatinine (Fig 2A). We further found that benzene induced dyslipidemia. Total cholesterol was elevated in benzene-exposed mice (120.0±7.1mg/dL) compared with mice breathing filtered air (107.0±4.7mg/dL) (Fig 2B). Benzene-exposed mice had higher plasma LDL (34.2±4.1 mg/dL) than mice breathing filtered air (25.6±3.3mg/dL) (Fig 2C). HDL levels in mice exposed to benzene (69.4±7.3 mg/dL) were 7.6% higher than in mice exposed to filtered air (64.5±4.6mg/dL) (Fig 2D). As a result, there was a 23% increase in the LDL:HDL ratio in benzene-exposed mice (Fig 2E). Triglyceride levels were unaffected (Fig 2F). Finally, benzene induced significant blood cell cytopenia (Table 3). Taken together, these results indicate that benzene inhalation in mice increased several indices commonly used to assess CVD risk in humans.

**Table 3:** Complete blood count of mice exposed to volatile benzene or HEPA-filtered air for six weeks.

Parameter		HEPA Mean (SD)	Benzene Mean (SD)	p-value	
**Leukocytes**					
** **	WBC (k/mL)	2.200 (0.782)	1.380 (0.099)	0.014	*
** **	NE (k/mL)	0.379 (0.140)	0.199 (0.099)	0.006	*
** **	LY (k/mL)	1.742 (0.641)	1.009 (0.246)	0.009	*
** **	MO (k/mL)	0.073 (0.040)	0.041 (0.015)	0.037	*
** **	EO (k/mL)	0.002 (0.004)	0.004 (0.008)	0.569	
** **	BA (k/mL)	0.003 (0.005)	0.002 (0.004)	0.628	
** **	NE (%)	17.57 (3.3)	14.59 (6.80)	0.234	
** **	LY (%)	79.16 (3.77)	81.49 (7.25)	0.382	
** **	MO (%)	2.92 (1.05)	3.39 (0.83)	0.282	
** **	EO (%)	0.106 (0.115)	0.041 (0.592)	0.215	
** **	BA (%)	0.146 (0.170)	0.000 (0)	0.024	*
**Erythrocytes**					
** **	RBC (M/mL)	8.52 (0.60)	7.10 (2.40)	0.098	
** **	HGB (g/dL)	11.6 (0.8)	9.7 (3.6)	0.126	
** **	HCT (%)	38.0 (2.5)	32.8 (11.7)	0.202	
** **	MCV (fL)	44.6 (0.8)	45.8 (2.1)	0.112	
** **	MCH (pg)	13.6 (0.5)	13.4 (0.7)	0.613	
** **	MCHC (g/dL)		30.5 (1.4)		29.4 (1.9)		0.146	
**Thrombocytes**						
	RDW (%)	17.2 (0.5)	16.7 (0.8)	0.135	
	PLT (k/mL)	768 (71)	601 (195)	0.028	*
	MPV (fL)	4.4 (0.1)	4.5 (0.1)	0.513	

*: p<0.05

n = 10 animals/treatment

### Human cohort characteristics

To directly assess associations between benzene exposure and cardiovascular health in humans, we enrolled a cohort of subjects with CVD or at risk of developing CVD (Table 1). The study cohort was middle-aged (51±10 years), with a slightly higher proportion of males (n = 109, 52%) and Whites (n = 118, 56%). The cohort included both current (n = 82, 39%) and former (n = 57, 27%) smokers. A majority of the participants was diagnosed with hypertension (n = 168, 81%), hyperlipidemia (n = 128, 63%), or obesity (body mass index ≥ 30, n = 118, 58%). Several participants were taking medications, including angiotensin converting enzyme (ACE) inhibitors (n = 112, 55%), beta-blockers (n = 129, 63%), and/or statins (n = 108, 53%). The median annual household income ($31,000) was lower than the median household income for Jefferson County of $46,298 [25].

The cohort was stratified into three *t*,*t-*MA levels (Table 1). Those stratified did not differ in age, gender, hypertension, diabetes mellitus, obesity, medical history, medication use, FRS, lymphocyte count, thrombosis, inflammation, or median household income. Compared with African Americans and Hispanic/Latinos, Whites were more likely to have higher *t*,*t-*MA levels (p = 0.001). Individuals who smoked or those with hyperlipidemia were more likely to have higher *t*,*t-*MA (p = 0.003, and p = 0.021, respectively). Cotinine levels were significantly higher in the high *t*,*t*-MA group than in the low *t*,*t*-MA group (p<0.001). Compared with low risk individuals (FRS < 20), high risk individuals (with FRS > 20), or those under secondary preventive care were more likely to have higher *t*,*t*-MA (p = 0.031).

### Association between *t*,*t*-MA and tobacco smoke exposure

To examine the link between tobacco smoke exposure and benzene, *t*,*t*-MA levels were regressed against cotinine, a metabolite of nicotine, and 3-HPMA, a metabolite of acrolein. The unadjusted regressions demonstrate positive associations between *t*,*t*-MA and both cotinine (p<0.001) and 3-HPMA (p<0.001) (Fig 1). On average, *t*,*t*-MA levels were 0.09% higher for every 1.0% increase in cotinine. In addition, the levels of 3-HPMA were 0.3% higher with 1.0% increase in cotinine levels, indicating a close association between smoking and acrolein exposure. Overall, *t*,*t*-MA was higher in smokers, suggesting that cigarette smoke is the major source of benzene exposure.

### Association between CACs and *t*,*t*-MA

Unlike mouse CACs which are defined by two markers, we used four markers in a flow cytometry-based strategy to identify 15 subtypes of human CACs [26]. To examine the association between benzene and these CAC subtypes, we constructed generalized linear models by regressing cell levels against *t*,*t-*MA (Table 2). After adjustment for ethnicity, hyperlipidemia, and cotinine, CAC-2 (CD31^+^/34^+^/45^+^), CAC-8 (CD31^+^/34^+^/45^+^/AC133^–^), and CAC-14 (CD34^+^/45^+^/AC133^+^) were significantly associated with *t*,*t-*MA. For each 0.1mg/g creatinine increase in *t*,*t*,-MA, there was a 8.7%, 8.9% and 9% decrease in CAC-, 2, 8 and 14, respectively. In non-smokers, after adjustment for ethnicity and hyperlipidemia, CAC-8 and CAC-14 were inversely associated with *t*,*t*-MA, indicating that even in non-smokers, exposure to benzene is associated with suppressed CAC levels.

Because *t*,*t*,*-*MA varied with ethnicity (Table 1), we stratified the cohort into Whites and African-Americans. After adjustment for hyperlipidemia and cotinine in Whites, CAC-2, 8 and 14 were inversely associated with *t*,*t*-MA. We estimate that for each 0.1 mg/g creatinine increase in *t*,*t*,-MA there was an 8.9% decrease in CAC-2, an 8.8% decrease in CAC-8, and an 8.4% decrease in CAC-14. Given that the demographic features of the African-Americans in the cohort were significantly different from the White population, a different set of adjustment factors was deemed more appropriate. After adjustment for gender, diuretics, calcium channel blockers, hypertension, revascularization, median household income, and cotinine, levels of cell type-2 (β = -1.444, p = 0.041), cell type-8 (β = -1.616, p = 0.029), and cell type-14 (β = -8.968, p = 0.001) were inversely associated with *t*,*t-*MA in the African-American population. In addition, *t*,*t*-MA levels were positively associated with cell type-11 (AC133^+^, β = 1.615, p = 0.009) within this population.

### Association of *t*,*t-*MA and CVD risk

**Fig 3:**
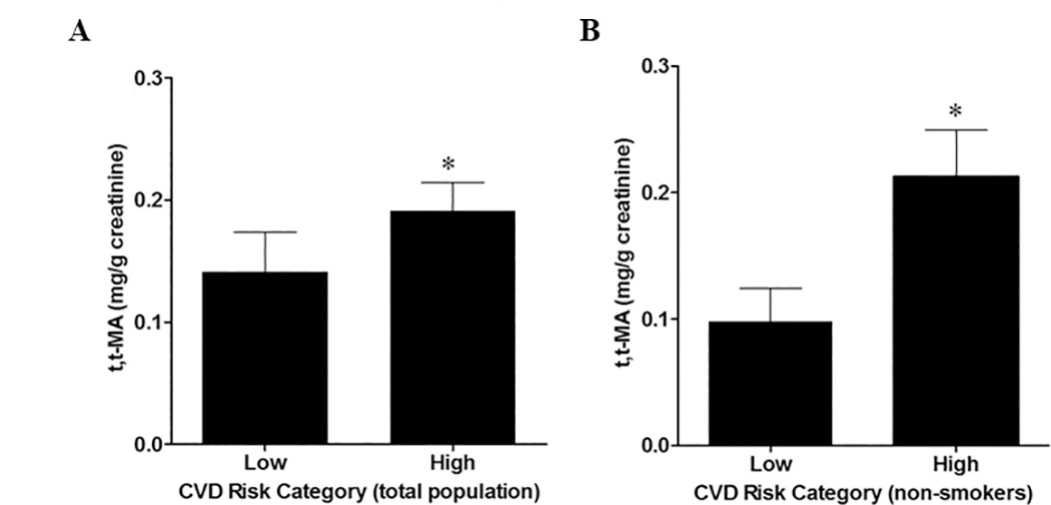
Association between *t*,*t-*MA and CVD risk. Associations between low (< 20; n = 44) and high (≥ 20 or experienced a cardiovascular event; n = 166) Framingham Risk Score and *t*,*t-*MA were determined for the total population (A; p = 0.024) and in non-smokers (B; p = 0.006).

To determine whether benzene exposure was associated with CVD risk, we stratified into low or high risk categories. Individuals with low CVD risk had significantly lower levels of *t*,*t-*MA than those in the high risk category (0.14±0.03 vs 0.19±0.03 mg *t*,*t*-MA /g creatinine) (Fig 3A). Because FRS estimates of CVD risk include smoking, this association may be partly due to benzene exposure from smoking. Hence, we examined the association between benzene exposure and *t*,*t*-MA. In non-smokers, we found that low CVD risk had significantly lower *t*,*t-*MA than those in the high risk category (0.10±0.03 mg *t*,*t*-MA/g creatinine vs. 0.21±0.04 mg *t*,*t*-MA/g creatinine) (Fig 3B). Plasma indices of inflammation and thrombosis, such as fibrinogen (p = 0.778), platelet leukocyte aggregates (p = 0.718), and hsCRP (p = 0.590) were not associated with *t*,*t*-MA or cotinine.

## Discussion

The major finding of this study is that benzene exposure is associated with increased cardiovascular disease risk and injury. This risk may be due in part to dyslipidemia and CAC pauperization as subjects in our human cohort with higher levels of *t*,*t*-MA had higher LDL levels and deficits of CACs which are indicative of vascular repair and predictive of cardiovascular events and mortality [16]. In addition we found that *t*,*t*-MA levels were associated with elevated CVD risk scores. Finally, while *t*,*t*-MA levels were higher in smokers than non-smokers, *t*,*t*-MA levels in non-smokers also showed a robust negative association with CAC levels. Collectively, these observations suggest that although smoking represents a major source of benzene exposure, exposure to benzene from other sources also likely induces cardiovascular injury and decreases vascular repair.

Although the health effects of occupational exposure to high levels of benzene have been studied, the effects of benzene at levels present in ambient air or tobacco smoke remain unclear. In addition, the contribution of benzene to the overall toxicity of tobacco smoke has not been assessed, and little is known about the cardiovascular effects of benzene. Our results showing that smokers had higher *t*,*t*-MA than non-smokers are consistent with previous reports and confirm that smoking is a major source of benzene [27, 28]. Indeed, in our analysis of smoking-derived metabolites (cotinine, *t*,*t*-MA and 3-HPMA), high *t*,*t*-MA was more closely associated with high cotinine than 3-HPMA (Fig 1B), suggesting that smokers and non-smokers could be more accurately distinguished by *t*,*t*-MA levels than 3-HPMA levels. This could be reflective of the fact that smoking is a major source of high level benzene exposure, whereas high level acrolein metabolites may arise from a variety of exogenous (e.g., food) or endogenous (e.g., myeloperoxidase or lipid peroxidation) sources [29–31]. Furthermore, our observation that *t*,*t*-MA was associated with CVD injury supports the notion that the harmful cardiovascular effects of smoking may be in part due to the presence of benzene. In our fully adjusted model, we found the association between *t*,*t*-MA and CACs was independent of cotinine. This may be due to differences in cigarette brand constituents or smoking behavior; differences that could lead to dissimilar levels of benzene exposure even at similar doses of inhaled nicotine.

Our observation that CVD risk was associated with *t*,*t*-MA in non-smokers suggests that benzene may be linked to cardiovascular injury, even in the absence of exposure to other tobacco smoke constituents; a view supported by our murine studies showing that exposure to benzene alone is sufficient to reduce vascular reparative cells. Hence, exposure to benzene, regardless of its source, and independent of the presence of other co-pollutants and toxicants, is predictive of cardiovascular injury and is associated with increased CVD risk. In non-smokers, we found that the *t*,*t*-MA ranged from 0.01 to 2.85 mg/g creatinine, which corresponds to an exposure range of benzene between 2 to 800 ppb [27]. The average ambient air concentration of benzene in most US cities is expected to range from 10 and 20 ppb, albeit higher levels could result from additional sources such as vehicle exhaust, gasoline, paints, adhesives, and solvents [4, 32–35]. Although exposure to high levels of benzene has been linked to acute myeloid and acute nonlymphocytic leukemias, low dose exposure (< 1 ppm) has been found to be associated with hematoxic effects and lymphohematopoietic (LH) cancers [23, 24]. Therefore, our observation showing an association between cardiovascular outcomes and benzene exposures corresponding to <1 ppm suggest that adverse CVD outcomes are at least as likely as LH cancers and hematoxicity in low dose exposures and may be more prevalent in the general population exposed to ambient benzene levels.

In both humans and mice we found that benzene exposure was associated with altered plasma lipoprotein levels. This finding suggests that dyslipidemia may be a significant outcome of benzene exposure, driving vascular inflammation and promoting the development of atherosclerotic lesions. Further cardiovascular risk derives from depletion of CACs, which are important for repair and regeneration of vascular tissue and are strongly associated with poor cardiovascular outcomes in susceptible individuals [16]. Previous studies have shown that CACs are highly sensitive to pollutant exposure as their abundance in circulation decreases upon exposure to tobacco smoke, acrolein and PM_2.5_ [15, 17, 36]. In the current study we found that benzene exposure was associated with low levels of specific CAC subpopulations (cell types 2, 8, 14) in humans and decreased Flk^+^/Sca-1^+^ cells in mice. Thus, these findings identify CACs as a new and sensitive target of benzene and suggest that both hematopoietic and non-hematopoietic stem cell populations can be affected by exposure. Given that both CVD risk and hematoxicity are sensitive to a similar range of benzene concentrations (*vide supra*), it seems likely that there might be a common locus of injury that affects primitive progenitor cells (i.e. hemangioblasts) that differentiate into both hematopoietic precursor and angiogenic progenitor cells. While further experiments are required to test this possibility, the high sensitivity of CD45^+^, but not CD45^-^ cells support the hypothesis that benzene exposure specifically affects hematopoietic cells and that depletion of CACs may result from the hematoxicity of benzene. The AC133^+^ cell population has been shown to enhance re‐endothelialization of vascular lesions when transplanted into mice and a 58% decrease in these cells predicted a 300% increase in CV mortality [16, 37]. Given this relationship, one might estimate that every 20 ppb increase in atmospheric benzene is likely to be associated with a 33% increase in CV mortality. Because benzene emissions associated with traffic pollution have been linked to the onset of acute myocardial infarction, our results suggest that benzene exposure, through suppression of CACs, may be a significant, and heretofore overlooked, CVD risk factor and pervasive trigger of acute cardiovascular events [38].

Our study has several strengths. It identifies a novel, inverse association between benzene and CACs in both humans and mice. Additional strengths of the study are assessments of individual exposure and risk indices, as exposure was estimated for each individual, based on urinary *t*,*t*-MA. Because blood and urine samples used to analyze CACs and *t*,*t*-MA were collected at the same time, we could concurrently evaluate both parameters which minimized mismatch due to temporal variations. Associations between *t*,*t*-MA and CACs were robust and even after correcting for multiple comparisons of 15 cell types, they remained significant (p<0.015). Moreover, the relationship between benzene and CVD risk was not obtained from a post‐hoc analysis of the data, but was examined to test an *a priori* hypothesis developed on the basis of previous data showing that exposure to benzene alters stem cell differentiation and growth [39–41]. This study also has some limitations. Although the cohort size is relatively large for one assessing CACs, it is fairly small for a study of CVD or environmental epidemiology. The cross-sectional design of the study is limited in its ability to determine causality. Moreover, the magnitude of benzene exposure varied. The effects of increased exposure may have been missed in a longitudinal prospective study if the exposure is not maintained throughout the observation period. However, the effects of simultaneous exposures are likely to be more apparent in a cross‐sectional design, although long-term trends may be difficult to interpret.

Although most *t*,*t*-MA arises directly from the metabolism of benzene by CYP2E1, it can also be generated at low levels by the metabolism of anti-microbial agents (e.g., sodium sorbate) which could lead to exposure misclassification. Another limitation of the study may be analysis from only a single urine sample from each subject. Therefore, our measurements may reflect a limited exposure. However, single time point urine collection samples correlate well with 24 h collection [42]. Circadian fluctuation in the expression of CYP2E1 or proximate environmental exposure were mitigated by collecting samples during the same time of day in the same laboratory [43]. Variability could also arise from individual differences in smoking behavior and metabolism of smoke constituents, which are not accounted for in our study. Genetic variation or dietary differences may alter CYP2E1 expression and activity, which would therefore alter the exposure to benzene metabolites [44].

Nevertheless, our results have wide implications as they suggest that exposure to benzene from any source could increase CVD risk. While a few studies have suggested a relationship between benzene exposure and insulin resistance or hypertension, these results have not been confirmed in animal models, or in well-controlled studies [45, 46]. Because benzene is a ubiquitous pollutant, further evaluation of its cardiovascular effects could inform exposure avoidance guidelines and regulatory policy to limit emissions by automobiles and industrial processes or to regulate tobacco products. Given the widespread exposure to benzene, even a modest decrease in exposure may substantially reduce the global burden of cardiovascular disease.

## Supporting information

S1 File Questionnaire given to study participants. (DOCX)
