# Supplementary material for: Benzene exposure is associated with cardiovascular disease risk
Source: PLoS One. 2017 Sep 8;12(9):e0183602. doi: 10.1371/journal.pone.0183602 (PMC5590846; doi:10.1371/journal.pone.0183602)
Supplement: S1 File — (DOCX) [file pone.0183602.s001.docx]

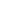


**Enrollment Questionnaire**

**EXPOSURE TO ENVIRONMENTAL ALDEHYDES AND CARDIOVASCULAR DISEASE RISK**

| Subject ID: |  | | | |
| --- | --- | --- | --- | --- |
| Subject Initials: |  | | | |
| Date: |  | | | |
| Permission to contact for future studies: |  | Yes |  | No |

1.

| Date of Birth: |  | Age: |  | (If less than 18 yrs, stop) |
| --- | --- | --- | --- | --- |

| Sex: |  | Height: | |  | | Weight: | lbs. | Race/Ethnicity: |  |
| --- | --- | --- | --- | --- | --- | --- | --- | --- | --- |
| Waist Circumference: | | |  | |  |  |  |  |  |

| Are you pregnant? |  | (If so, stop) |  |  |  |
| --- | --- | --- | --- | --- | --- |
| When was your last menstrual cycle (if applicable)? | | | |  |  |

2.

| Address: |  | | (street address) |  |
| --- | --- | --- | --- | --- |
|  |  | | (city, state, zip) |  |
| How long have you lived at your current address? | |  | | |

3.

Do you have any history of:

| Heart attack? |  | Yes |  | No. | | If yes, when? | | |  | | |
| --- | --- | --- | --- | --- | --- | --- | --- | --- | --- | --- | --- |
| Heart failure? |  | Yes |  | No. | | If yes, when? | | |  | | |
| Heart bypass surgery,  angioplasty, or heart stents? |  | Yes |  | | No. | | If yes, when? | | |  | |
| Angina? |  | Yes |  | No. | |  | | | | |  |
| High blood pressure? |  | Yes |  | No. | |  | | | | |  |
| High blood cholesterol? |  | Yes |  | No. | |  | | | | |  |
| Diabetes? |  | Yes |  | No. | |  | | | | |  |
| Stroke? |  | Yes |  | No. | | If yes, when? | | |  | | |
| Irregular heart rhythms? |  | Yes |  | No. | | If yes, when? | | |  | | |
| Poor circulation in the legs? |  | Yes |  | No. | |  | | | | |  |
| Aortic aneurysm? |  | Yes |  | No. | | If yes, when? | | |  | | |
| Bleeding disorders? |  | Yes |  | No. | |  | | | | |  |
| Clotting? |  | Yes |  | No. | |  | | | | |  |
| Oral contraceptive use? |  | Yes |  | No. | |  | | | | |  |
| Other? |  | Yes |  | No. | | Specify: | |  | | | |

4.

| Do you currently smoke? | | |  | | Yes | |  | | | No. | | If yes, how much do you smoke? | | | | | | | | ppd | | |  |
| --- | --- | --- | --- | --- | --- | --- | --- | --- | --- | --- | --- | --- | --- | --- | --- | --- | --- | --- | --- | --- | --- | --- | --- |
| Never smoked: | | |  | | (Less than 1 pack of cigarettes in lifetime) | | | | | | | | | | | | | | | | | | |
| Former smoker: | | |  | | How much did you smoke? | | | | | | | | | | | | ppd | | When did you stop? | |  | |  |
| Do you use other tobacco products? | | | | | |  | | | If yes, please list: | | | | | | |  | | | | | | |  |
| Do you use electronic cigarettes? | | | | | |  | | | Yes | |  | | | No. |  |  |  |  |  |  |  |  |  |
| Are you exposed to secondhand smoke in any of the following environments? | | | | | | | | | | | | | | | | | | | | | |  |  |
| Work: |  | Home: | |  | | | | School: | | | | |  | | | | |  |  |  |  |  |  |

5.

| Do you drink alcohol? |  | If yes, how much alcohol do you drink per week? |  |
| --- | --- | --- | --- |

6.

| Do you exercise regularly? |  | Yes |  | No. | If yes, what type of exercise and how often? |
| --- | --- | --- | --- | --- | --- |
|  | | | | | |

7.

| If working, what is your occupation? |  |
| --- | --- |

| Address of employment? |  | | (street address) |
| --- | --- | --- | --- |
|  |  | | (city, state, zip) |
| How long have you worked there? | |  |  |

| Have you spent in the following workplace environments? If yes, how much time? | | | | | | | | | |
| --- | --- | --- | --- | --- | --- | --- | --- | --- | --- |
|  | Factory |  | Office |  | Construction |  | Outdoor machinery |  |  |
|  | Agriculture |  | Transportation |  | Hospital |  | Restaurant |  |  |
|  | Janitorial |  | Other specify: |  | | | | |  |

| What is your education level? 🞎 <High School 🞎 High School/GED 🞎 Some College |
| --- |
| 🞎 2-yr Degree 🞎 4-yr Degree 🞎 Master’s 🞎 Doctoral |

| What is your mother’s education level? 🞎 <High School 🞎 High School/GED |
| --- |
| 🞎 Some College 🞎 2-yr Degree 🞎 4-yr Degree 🞎 Master’s 🞎 Doctoral 🞎 Unsure |

| What is your annual household income (last 5 years)? 🞎 <$20,000 🞎 $20,000-$44,999 | |
| --- | --- |
| 🞎 $45,000-$64,999 🞎 $65,000-$89,999 🞎 $90,000-$124,999 🞎 >$125,000 |  |

8.

| What is the amount of time you spend indoors each day? | | |  |
| --- | --- | --- | --- |
| What is the age of your home? | years |  |  |

9.

| List all over-the-counter supplements and prescribed medication you are currently taking: |
| --- |
|  |
|  |
|  |
|  |
